# Application of orthopedic robotics in cancer surgery: integrating precision treatment and postoperative exercise rehabilitation

**DOI:** 10.3389/fonc.2026.1784633

**Published:** 2026-10-19

**Authors:** Jing Wang, Qin Xuan, Zhenyu Yang, Yang Wu, Yushen Xu, Menghao Tang, Yihan Wang, Xinming Ye, Mei Du

**Affiliations:** 1 School of Sports Science and Engineering, East China University of Science and Technology, Shanghai, China; 2 Medical College, Ningde Normal University, Ningde, China

**Keywords:** bone cancer surgery, cancer surgery, clinical application, orthopedic robotics, physical rehabilitation, soft tissue tumor resection

## Abstract

This article provides a narrative review of selected applications of orthopedic robotics in cancer surgery, focusing on musculoskeletal tumor resection, relevant technical developments, and implications for postoperative rehabilitation. Orthopedic robotic technology has evolved continuously, with advances in image guidance, instrument positioning, and operative planning. Robotic assistance may improve the reproducibility of complex osteotomies and reconstruction, but technical accuracy should be distinguished from demonstrated oncologic or functional benefit. Current evidence includes experimental models, case reports, and navigation-assisted series, which do not establish that robotic surgery reduces recurrence or accelerates recovery after orthopedic tumor surgery. Beyond intraoperative applications, artificial intelligence and digital rehabilitation platforms offer potential ways to use surgical data, baseline function, and activity measures to inform individualized rehabilitation and follow-up. Rehabilitation robots, including exoskeletons, represent a separate technology whose suitability and benefits require evaluation in specific patient populations. Integration of these approaches into orthopedic oncology remains a direction for clinical research. Ethical and legal challenges, cost-effectiveness, and unequal access also require consideration. This review highlights the potential of orthopedic robotics while identifying the need for comparative studies of resection margins, long-term tumor control, functional recovery, and quality of life.

## 1 Introduction

Robotic technology has become an increasingly investigated tool for image-guided planning and instrument positioning in orthopedic surgery (1). This narrative review explores its application and development in cancer surgery, focusing on musculoskeletal tumor resection, associated reconstruction, and rehabilitation. Here, orthopedic robotics refers to systems that guide or assist a surgical task. Computer-assisted navigation and patient-specific guides are related technologies but do not necessarily involve a robot; postoperative rehabilitation devices serve a separate function. Initially introduced in neurosurgery during the 1980s, robotic systems have evolved from assisting with simple tasks to supporting increasingly complex procedures (2). Real-time navigation, minimally invasive approaches, and AI integration have been investigated in spinal and joint surgeries (3). Evidence on bone tumor resection also includes patient-specific instrumentation and surgical navigation, and should not be interpreted as proof of superior robotic resection or faster recovery (4). Tuoshou and TiRobot are image-guided positioning and navigation systems, described further in Section 4.1. AI-assisted rehabilitation is another developing application (5), whose integration with orthopedic tumor surgery requires validation. However, challenges such as ethical considerations, legal responsibility, and financial barriers remain. Continued research is essential to evaluate long-term oncological outcomes and to ensure equitable access to this evolving technology.

This narrative review uses a bibliography spanning 2013–2026 to discuss selected clinical applications and associated technologies. During revision, sources were assessed qualitatively for relevance to musculoskeletal tumor surgery or rehabilitation, study design, population, outcomes, and limitations. Direct clinical evidence was distinguished from experimental studies and cross-specialty background. Key existing studies were verified by title or DOI in Europe PMC on 10 September 2026; two additional rehabilitation and follow-up publications were verified on 14 September 2026. This targeted verification was not an exhaustive database search. No systematic search strategy, predefined systematic eligibility criteria, or formal risk-of-bias assessment was applied. Relevant studies may therefore have been omitted, and the review does not provide an exhaustive synthesis or clinical practice recommendations.

## 2 Fundamental theories of orthopedic robotics in cancer surgery

### 2.1 Evolution of orthopedic robotic technology

The development of orthopedic robotics has progressed through cumulative innovations in imaging, robotics, and computer science. Since its introduction by Professor Kwoh in 1985, the scope of robotic-assisted surgery has expanded beyond neurosurgery into orthopedics and rehabilitation (6).Early systems provided basic assistance, but recent generations enable complex interventions, especially in spinal procedures where precision is critical (7).Innovations in 3D imaging have improved surgical planning and intraoperative navigation. Second-generation spinal robots have addressed early technical limitations, enhancing both precision and safety, particularly in minimally invasive surgeries (8).Intelligent navigation systems now support real-time adjustments, contributing to the widespread adoption of robotics in spine operations. In rehabilitation, robotic systems have also been evaluated for upper limb recovery in post-stroke patients (9). These systems facilitate accurate movement control, individualized treatment, and real-time feedback, reflecting the convergence of imaging, AI, and motor control in modern orthopedic care.

### 2.2 Application principles of orthopedic robotics in cancer surgery

Robotic assistance is based on image guidance, instrument control, and the integration of surgical information (10). Preoperative CT and MRI can support anatomical planning, while navigation relates the planned trajectory to the operative field. Navigation has also been studied in robotic-assisted and laparoscopic partial nephrectomy (11), providing cross-specialty context rather than direct evidence for orthopedic tumor resection. Some systems include force-sensing and feedback capabilities to inform tissue interaction (12). These features are relevant to work near delicate structures, but their clinical value depends on registration accuracy, instrument performance, and the surgical task. In musculoskeletal oncology, technical capabilities alone do not establish fewer complications, adequate tumor margins, or earlier mobilization.

### 2.3 Comparison between robotic-assisted and traditional surgical methods

Compared with traditional approaches, robotic-assisted procedures offer technical advantages in selected indications, but their clinical effects depend on the procedure and comparator. Reports in oral and maxillofacial surgery illustrate the use of robotics in confined anatomical spaces (13). Comparative studies have also evaluated outcomes after robotic-assisted total knee arthroplasty (14) and percutaneous screw fixation for calcaneal fractures (15). These studies provide background on precision, workflow, and recovery in their respective populations. Their findings should not be transferred directly to orthopedic tumor patients, whose outcomes also depend on tumor extent, resection margins, reconstruction, and additional treatment. Improved positioning or osteotomy accuracy therefore does not by itself establish shorter hospitalization, faster rehabilitation, or superior tumor control after musculoskeletal cancer surgery.

## 3 Clinical practices of orthopedic robotics in cancer surgery

### 3.1 Application cases of orthopedic robotics in bone cancer surgery

Robotic platforms have expanded the technical options available in orthopedics (16), although the evidence relevant to bone tumor surgery includes different technologies and study designs. Khan et al. compared haptic robot-assisted and manual simulated resections in 12 synthetic femora, with six specimens per group. Compared with manual cutting, the robotic technique produced mean improvements of 7.8 mm in maximum linear deviation, 7.9° in pitch, and 4.6° in roll (17). This experimental study demonstrated improved resection accuracy but did not assess clinical complications or cancer outcomes. Eroglu et al. reported transoral robotic resection of an odontoid metastasis in a 48-year-old woman after occipitocervical fixation. The patient was discharged in good condition, but this single case cannot establish comparative safety or recovery benefits (18). Gerbers et al. described 130 computer-assisted orthopedic oncology procedures involving navigation for intralesional treatment, resection, and reconstruction, and reported a high proportion of R0 resections (19). However, the heterogeneous procedures and descriptive study design do not establish that robotic surgery improves margin status or reduces recurrence compared with conventional surgery.

### 3.2 Technical challenges in soft tissue tumor excision

Force control is a relevant technical issue when robotic instruments operate near delicate tissues. Xia et al. developed a framework using viscoelastic tissue modeling and force optimization for orthopedic surgical robots, with validation through simulation (20). The work provides an experimental basis for controlling tissue interaction during tasks such as laminectomy. It does not demonstrate clinical effectiveness in soft tissue sarcoma excision. Application to deformable tumor tissue requires further validation of localization, force control, and tissue safety.

### 3.3 Postoperative recovery and complication management in orthopedic robotic surgery

Postoperative rehabilitation is an important component of care, but evidence that robotic surgery itself accelerates recovery after orthopedic tumor resection remains limited. An enhanced recovery study in pancreatoduodenectomy associated robotic surgery with early discharge (21); this provides indirect perioperative context rather than evidence for musculoskeletal tumor patients. Separately, a recent review discusses robotic exoskeletons as motion-assistive systems in cancer rehabilitation, including assisted movement and individualized training (64). This is an emerging rehabilitation application, not a demonstrated benefit of the robot used for tumor resection. Rehabilitation planning should reflect the individual patient’s functional status and clinical restrictions (65), while the safety and long-term value of exoskeleton use after orthopedic tumor surgery require dedicated evaluation. Preclinical research also suggests that exercise-induced circulating exosomes may alleviate chemotherapy-induced peripheral neuropathy by suppressing microglial PHLDA1 and neuroinflammation (66).

Complication management also requires attention. Reviews of transoral robotic procedures and spinal image guidance discuss issues such as bleeding, nerve injury, and radiation exposure (22, 23). These risks arise in different surgical settings and should not be pooled as evidence of faster rehabilitation after orthopedic tumor resection. The evidence summarized here is insufficient to establish a rehabilitation advantage attributable to surgical robotics in this population. Prospective studies should assess functional recovery, complications, and patient-reported outcomes alongside resection quality.

## 4 Technological advancements in orthopedic robotics for cancer surgery

### 4.1 Recent advancements in orthopedic robotic navigation systems

Innovations in robotic navigation have enhanced instrument positioning. Tuoshou integrates the robotic arm and workstation and incorporates an integrated tracker and guide assembly. TiRobot comprises a robotic arm, an optical tracking device, and a surgical planning and control workstation. In a randomized comparison, Tuoshou achieved better results on selected Kirschner wire and pedicle screw placement measures, including smaller entry-point deviation (p < 0.001) (24). Several other deviation measures and safety indicators did not differ significantly. This trial assessed spinal instrumentation in a mixed surgical population rather than tumor resection or rehabilitation outcomes. Robotic navigation has also been investigated in intra-articular lower-limb fracture surgery (25), and a meta-analysis evaluated robot-assisted pedicle screw placement in lumbar spondylolisthesis (26). These studies provide technical and non-oncologic context, but do not establish improved orthopedic cancer outcomes.

### 4.2 Innovations in the application of orthopedic robotics in minimally invasive surgery

Robotic systems support minimally invasive instrument positioning in several surgical settings. Minimally invasive robotic spine surgery illustrates an instrumentation workflow (27), while experience with the da Vinci single-port system in thoracic surgery provides cross-specialty context (28). In a retrospective pelvic fracture study, adding preoperative 3D printing to robotic screw fixation increased the proportion of screws without cortical breach from 63.3% to 88.5%, compared with robotic fixation alone (29). Robot-guided femoral neck fracture fixation also uses infrared tracking and a robotic arm to support planned screw placement (30). These examples concern instrumentation or other surgical specialties and do not establish superior tumor resection or functional recovery in orthopedic oncology.

### 4.3 Integration of orthopedic robotics with artificial intelligence

Artificial intelligence (AI) is being investigated for data-driven orthopedic planning and personalized care (31). Analysis of imaging data may support preoperative planning and surgical decision-making (32). In spine surgery, current applications include planning and prediction (33), while orthopedic imaging and research offer additional areas for evaluation (34). Digital follow-up is a related but distinct component: SportSync Health records functional assessments and supports longitudinal follow-up and clinician–patient communication in sports medicine (50). This report illustrates a possible framework for collecting recovery data; it does not validate an AI-controlled rehabilitation pathway in orthopedic oncology. Integrating such information with robotic surgical planning may support individualized care, but benefits in tumor control, functional recovery, and efficiency require prospective clinical evaluation.

## 5 Clinical context and oncologic considerations

### 5.1 Epidemiological features and surgical demands of bone cancer

Primary bone malignancies are heterogeneous in histology and treatment requirements, with rare subtypes posing additional diagnostic challenges (35). Osteosarcoma mainly affects children and adolescents, and its treatment incorporates surgery and systemic chemotherapy (36). Ewing sarcoma also commonly affects young patients and requires multimodal treatment, while metastatic or relapsed disease remains associated with poor outcomes (37). Population-based research on skull-base chondrosarcoma has examined incidence and survival in this specific anatomical setting (38), and these findings should not be generalized to all chondrosarcomas. These differences highlight the need for tumor-specific surgical planning. The role of robotic assistance should therefore be evaluated for defined surgical tasks and patient populations, with technical accuracy distinguished from improvements in prognosis or recovery.

### 5.2 Resection margins and oncologic outcomes

The studies discussed here primarily address technical performance and surgical outcomes rather than direct effects on tumor biology. Reviews of robotic oncology describe technological developments and potential clinical applications (39), while head and neck surgery provides examples of advances in robotic access and surgical visualization (40). In musculoskeletal oncology, the reports discussed in Section 3.1 illustrate technical feasibility and margin assessment, but do not establish comparative oncologic superiority. Geometric accuracy, R0 resection, local recurrence, and survival are distinct endpoints. Claims that robotic assistance improves tumor control therefore require comparative studies with adequate follow-up and consideration of tumor characteristics and additional treatment.

### 5.3 Potential applications in orthopedic oncology

Potential applications in orthopedic oncology include selected tasks in musculoskeletal tumor resection and associated reconstruction. Robotic pedicle screw placement in spine surgery provides relevant instrumentation experience (41). Transoral surgery for early-stage oropharyngeal cancer (42), a pediatric tumor series (43), and robotic rectal surgery (44) illustrate applications in other oncologic settings. These examples provide contextual experience rather than established indications for orthopedic tumor surgery. Suitability depends on the surgical task, anatomical access, imaging, and registration requirements. Feasibility in another anatomical region does not by itself establish safety or oncologic benefit in bone or soft tissue tumors.

## 6 Orthopedic robotics in cancer surgery: diagnosis and treatment strategies

### 6.1 Image-guided localization and surgical planning

Image guidance supports anatomical localization and operative planning rather than establishing a histopathological diagnosis. Computer-assisted planning has been described for midfacial tumor resection and reconstruction (45), while comparative evidence in femoral neck fractures concerns screw placement (46). These are different clinical applications. For bone tumor resection, patient-specific instrumentation and surgical navigation provide more directly relevant planning evidence (4). Their contribution should be assessed through resection accuracy and appropriate clinical endpoints; neither better localization nor improved screw placement alone establishes higher R0 rates or faster functional recovery.

### 6.2 Optimization of treatment strategies with orthopedic robotics in cancer surgery

Image-based planning and trajectory guidance can support the execution of selected surgical tasks. Patient-specific instrumentation and navigation have been evaluated in bone tumor resection (4). In minimally invasive lumbar fusion, robotic navigation has also been compared with navigation alone for pedicle screw selection and accuracy (47). This instrumentation study does not evaluate tumor excision. Translating planning tools to oncologic procedures requires attention to registration and the intended resection margin, with technical accuracy assessed separately from cancer outcomes.

### 6.3 Personalized treatment plans in orthopedic robotic surgery

Orthopedic robotics can support individualized planning using patient-specific anatomical data (48). Personalized arthroplasty approaches aim to account for native anatomy and joint function (49), while 3D printing combined with robotic pelvic fracture fixation provides another example of patient-specific planning (29). These applications arise from non-oncologic practice and offer technical context for musculoskeletal tumor surgery. Their effects on resection margins, complications, and postoperative recovery in orthopedic oncology require direct clinical evaluation.

## 7 Controversies and future prospects of orthopedic robotics in cancer surgery

### 7.1 Ethical and legal challenges in orthopedic robotic surgery

The increasing adoption of orthopedic robotic systems in cancer surgery has raised significant ethical and legal concerns (51). A primary ethical issue involves ensuring valid informed consent. Given the complexity of robotic procedures, patients often struggle to fully comprehend the associated risks and mechanisms, potentially compromising their autonomy (52).Particularly in complex oncology cases, decisions may be swayed more by medical authority than by patient understanding (53).It is essential to establish robust mechanisms to ensure patients make voluntary, informed decisions.

Another ethical dilemma concerns accessibility. The high cost of acquiring and maintaining robotic systems may limit their availability to wealthier patients, raising concerns about healthcare equity (54).Ensuring equitable access across socioeconomic groups remains a pressing challenge, requiring strategies that broaden the availability of robotic surgery regardless of financial background.

From a legal standpoint, assigning responsibility in robotic-assisted procedures is increasingly complex. Beyond the traditional liability of surgeons and hospitals, robotic system manufacturers may also be held accountable in cases of malfunction or design flaws (55).Telesurgery adds further complications, including jurisdictional discrepancies and cybersecurity risks (56).Therefore, establishing a comprehensive legal framework is critical to delineate responsibilities, protect patient rights, and support innovation.

### 7.2 Cost-effectiveness analysis of orthopedic robotics in cancer surgery

Evaluating the cost-effectiveness of robotic systems in cancer-related orthopedic surgeries is essential. Although robotic technologies may improve technical precision, their high upfront costs pose challenges. Reviews of spinal robotics evaluate accuracy, adverse events, and costs (57), but these findings do not establish cost-effectiveness in orthopedic tumor surgery.

In rectal cancer surgery, studies have shown that while robotic surgery incurs higher initial costs than laparoscopy, overall treatment costs may not differ significantly (58). Economic evaluations in thoracic and abdominopelvic surgery commonly use quality-adjusted life years, with cost-effectiveness estimates depending on the indication, analytical perspective, time horizon, and surgical volume (59).However, some findings highlight that robotic rectal resections incur higher total costs when including rehospitalizations and related expenses, limiting adoption in cost-sensitive settings (60).

In gynecological cancer surgeries, fixed system costs are the primary contributors to the elevated expenses (61). Experience from robotic bariatric surgery also links surgical volume and team learning to operating-room efficiency (62). These cross-specialty findings inform implementation questions, but orthopedic oncology requires its own economic evaluation because procedure volume, complexity, and follow-up needs differ.

### 7.3 Future development directions of orthopedic robotics in cancer surgery

Technological advancements continue to shape the future of orthopedic robotics in oncology. One focus is enhancing tactile feedback systems to improve intraoperative sensitivity to tissue characteristics, thereby increasing surgical accuracy (63). The integration of artificial intelligence (AI) may support surgical planning, intraoperative decision-making, and the interpretation of recovery data. Combining surgical robotics with individualized rehabilitation is a research direction; whether this integration accelerates recovery after orthopedic tumor surgery remains to be established.

Clinical translation is constrained by learning curves, additional workflow demands, registration or tracking errors, instrument deviation, and the possible need to revert to conventional techniques (23, 41, 57). Navigation depends on accurate correspondence between imaging and the operative anatomy. Soft-tissue deformation presents an additional challenge when extending methods designed for rigid bone, while force control remains an important area of experimental investigation (20). Case reports, experimental models, and uncontrolled series cannot establish superiority over conventional surgery. Future comparative studies should assess resection margins, local recurrence, survival, and functional recovery alongside geometric accuracy, with adequate follow-up to determine whether technical improvements translate into patient benefit.

Personalized medicine is another promising avenue. Future robotic systems may tailor surgical plans to individual tumor characteristics and patient anatomy. Whether such approaches reduce complications or improve quality of life and survival requires prospective evaluation.

Widening access to robotic surgery remains a key goal. This will require further cost reductions through technological innovation, scale production, and healthcare resource optimization. Expanding the availability of robotic systems in low-resource settings will help reduce disparities and extend the benefits of robotic oncology surgery to a broader patient population.

## 8 Conclusion

Orthopedic robotics offers technical opportunities for image-guided planning, instrument positioning, and selected tasks in tumor resection and reconstruction. This narrative review has outlined the relevant technologies and clinical reports while distinguishing surgical robotics from navigation and rehabilitation devices. Experimental improvements in precision and reported clinical feasibility are promising, but evidence for superior long-term tumor control or functional recovery over conventional surgery remains limited.

Postoperative rehabilitation remains an important component of care. Robotic exoskeletons and digital follow-up platforms offer potential tools for assisted movement and functional assessment, but their integration with orthopedic tumor surgery requires direct evaluation. Future studies should distinguish the effects of surgical assistance from those of rehabilitation interventions and assess their suitability for specific patient populations.

Widespread implementation also faces ethical, legal, and financial barriers, including informed consent, responsibility, training, and unequal access. Future development should pair technical refinement with comparative studies of resection margins, complications, long-term oncologic outcomes, and patient-reported function. Collaboration among surgeons, rehabilitation professionals, engineers, and other stakeholders will be essential to evaluate these technologies and guide their appropriate use.
